# Clinical applications of circulating tumor-derived DNA in the management of gastrointestinal cancers – current evidence and future directions

**DOI:** 10.3389/fonc.2022.970242

**Published:** 2022-09-29

**Authors:** Rachel C. T. Lam, David Johnson, Gigi Lam, Michelle L. Y. Li, Joyce W. L. Wong, W. K. Jacky Lam, K. C. Allen Chan, Brigette Ma

**Affiliations:** ^1^ Faculty of Medicine, The Chinese University of Hong Kong, Hong Kong, Hong Kong SAR, China; ^2^ Department of Clinical Oncology, State Key Laboratory of Translational Oncology, Sir Y. K Pao Centre for Cancer, Hong Kong Cancer Institute, Prince of Wales Hospital, The Chinese University of Hong Kong, Hong Kong, Hong Kong SAR, China; ^3^ Department of Chemical Pathology, The Chinese University of Hong Kong, Hong Kong, Hong Kong SAR, China

**Keywords:** ctDNA, gastrointestinal cancer, minimal residual disease, prognostic and predictive biomarker, next generation sequencing

## Abstract

Advances in Next Generation Sequencing (NGS) technologies have enabled the accurate detection and quantification of circulating tumor-derived (ct)DNA in most gastrointestinal (GI) cancers. The prognostic and predictive utility of ctDNA in patiets with different stages of colorectal (CRC), gastro-esophageal (GEC) and pancreaticobiliary cancers (PBC) are currently under active investigation. The most mature clinical data to date are derived from studies in the prognostic utility of personalized ctDNA-based NGS assays in the detection of minimal residual disease (MRD) and early recurrence after surgery in CRC and other GI cancers. These findings are being validated in several prospective studies which are designed to test if ctDNA could outperform conventional approaches in guiding adjuvant chemotherapy, and in post-operative surveillance in some GI cancers. Several adaptive studies using ctDNA as a screening platform are also being used to identify patients with actionable genomic alterations for clinical trials of targeted therapies. In the palliative setting, ctDNA monitoring during treatment has shown promise in the detection and tracking of clonal variants associated with acquired resistance to targeted therapies and immune-checkpoint inhibitors (ICI). Moreover, ctDNA may help to guide the therapeutic re-challenge of targeted therapies in patients who have prior exposure to such treatment. This review will examine the most updated research findings on ctDNA as a biomarker in CRC, GEC and PBCs. It aims to provide insights into how the unique strengths of this biomarker could be optimally leveraged in improving the management of these GI cancers.

## 1 Introduction

According to GLOBOCAN 2020, several gastrointestinal (GI) cancers are amongst the top ten most prevalent and lethal cancers in certain parts of the world (1). Colorectal cancer (CRC) accounts for one in every 10 cancer-related deaths and is most prevalent in Western countries. Gastric cancer (GC) and esophageal squamous cancer (ESCC) are more common in East Asia and are responsible for one in every 13 and one in 18 cancer-related deaths in the world, respectively. The overall incidence rates of CRC, pancreatic cancer (PC) and biliary cancers (BLC) are stable or declining, but GC and esophageal adenocarcinoma (GEA) show rising trends in younger people from developed countries (1). Systemic therapy is integral to the management of some advanced GI cancers and the use of biomarkers in guiding treatment decisions may improve patient’s outcome (2–4).

**Figure 1:**
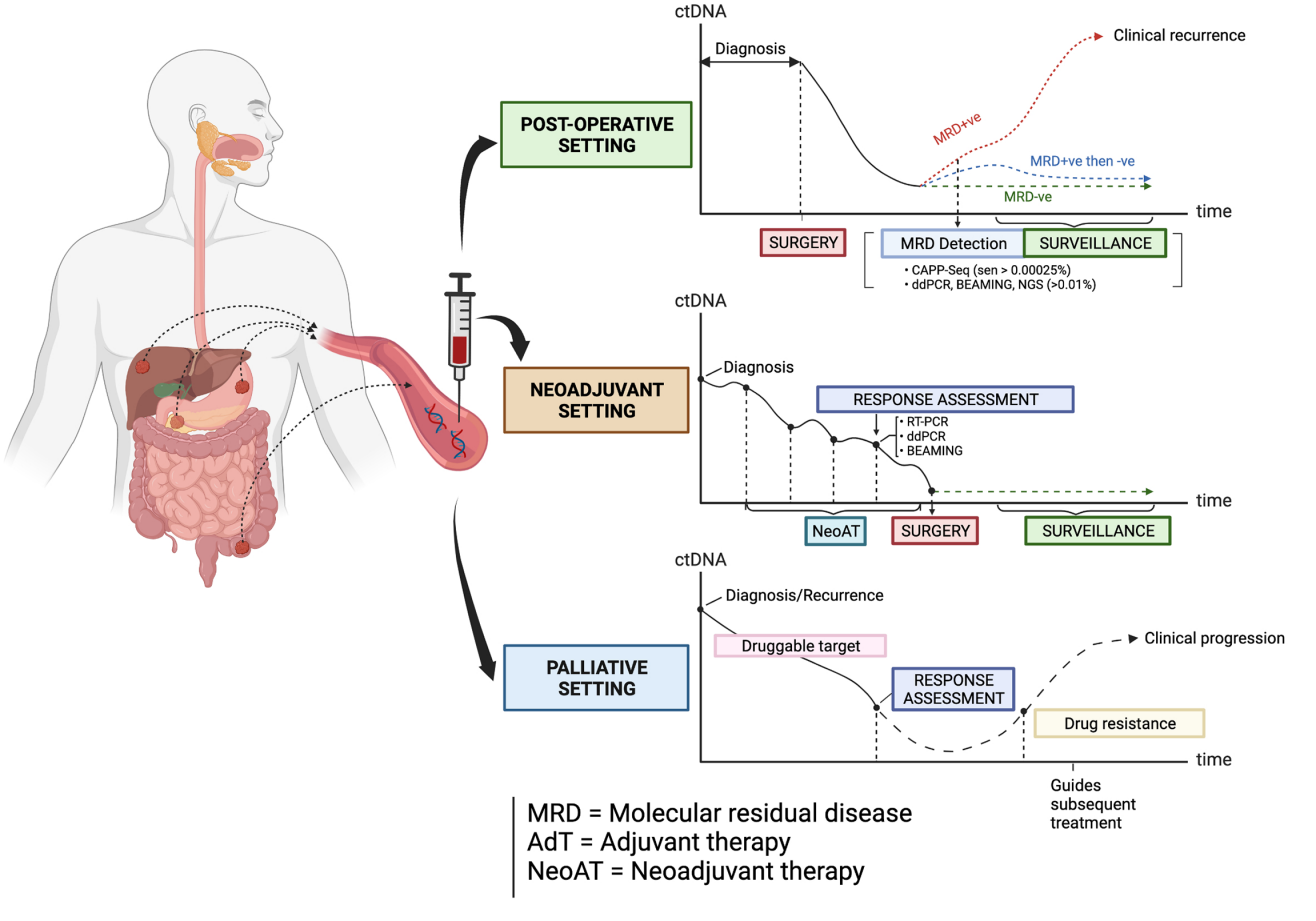
The types of clinical settings where ctDNA is being investigated as a biomarker in guiding the management of some gastrointestinal cancers. Created with BioRender.com.

Circulating tumor DNA (ctDNA) is a non-invasive and promising biomarker which is under active investigation in patients with GI cancers. The term ‘liquid biopsy’ refers to the process of sampling ctDNA, which is a component found in cell-free DNA (cfDNA) originating from the direct release, active secretion, necrosis or apoptosis of tumor cells into the circulation (5, 6). Each fragment of ctDNA usually has an average size of 166 base pairs, which resembles mononucleosomal units originating from cellular apoptosis (7). In recent years, research studies have evaluated the utility of ctDNA in the management of some GI cancers in these clinical settings: 1) the detection of minimal (or molecular) residual disease (MRD) following surgical resection of the primary tumor and in guiding adjuvant therapy; 2) assessment of clinical response to neoadjuvant chemotherapy and/or radiotherapy; 3) monitoring of response to palliative drug therapies; 4) tracking of clonal dynamics and evolution during targeted therapy, as well as in 5) the enrichment and selection of patients for clinical trials of novel anti-cancer therapies (**Figure 1**). The main objective of this article is to review the latest and most salient research studies on the clinical application of ctDNA in patients with advanced CRC, GEA, ESCC, PC and BLC. This review will also focus on how the strengths of ctDNA can be optimally leveraged in improving the treatment of these GI cancers.

## 2 Overview of ctDNA as a biomarker in gastrointestinal cancers

The quantification of ctDNA in solid tumors generally involves two broad categories of assays: tumor-informed and tumor-agnostic assays (8, 9). Tumor-informed assays require a prior knowledge of tumor-specific genomic alterations. One of the commonest platforms used is the polymerase chain reaction (PCR)-based assays, which include droplet digital PCR (ddPCR), quantitative real-time (RT-qPCR) and ‘Beads, Emulsion, Amplification and Magnetics’ (BEAMing) PCR (8, 9). Another type of platform is to apply Next-Generation Sequencing (NGS) on a target panel of genomic alterations, examples of which include the Tagged-Amplicon deep sequencing (TAm-seq), Safe-sequencing System (Safe-SeqS) and CAncer Personalized Profiling by deep sequencing (CAPP-Seq) (8, 10). Such NGS-based assays are highly sensitive with a Limit of Detection (LOD) of variant allelic frequencies (VAF) as low as 0.01%, and specific in detecting various mutations including indels, rearrangements and copy number alterations (CNAs) in GI cancers. In contrast, tumor-agnostic assays are broad, panel-based sequencing assays that detect genomic alterations and methylation changes (9). They allow real-time tracking of novel mutational changes and cancer-specific variants simultaneously (8, 9).

In general, the detection rates of ctDNA can vary between different types of GI cancers. Bettegowda et al. found that ctDNA could be detected in around 73%, 57% and 48% of patients with CRC, GEC and PC, respectively (11). Strickler et al. reported a high correlation between the rates of ctDNA-derived and tumor-derived NGS-based detection of 20 most commonly mutated genes in CRC (12). However, the detection and interpretation of ctDNA are potentially limited by several patient-related and assay-related factors. Discordance between tumor and plasma samples may be influenced by intra-tumoral heterogeneity, tumor histology, anatomical location of metastases and the patient’s tumor burden. For instance, GC has a higher level of genomic heterogeneity than PC and CRCs, resulting in more variable interpatient rates of ctDNA detection. The level of tumor DNA shedding into plasma is lower with mucinous tumors and locoregional metastases, compared with liver metastases in CRC (11, 13, 14).

Limitations resulting from these pre-analytical and assay-related factors may undermine the accuracy of ctDNA results. False-negative results may be caused by the low VAF of specific variants or from inadequate volumes of plasma sampled (9). Since cfDNA is also released by blood cells, the expansion of blood cells in clonal hematopoiesis of indeterminate potential (CHIP) may increase the level of background noise signals and false-positive ctDNA measurements (15). According to a consensus statement by the National Cancer Institute (NCI) Colon-Rectal-Anal Taskforce, limitations related to ctDNA assays could be minimized by standardization of a common protocol for blood collection, sample processing, DNA extraction and analysis (16).

## 3 Colorectal cancer

**Figure 2:**
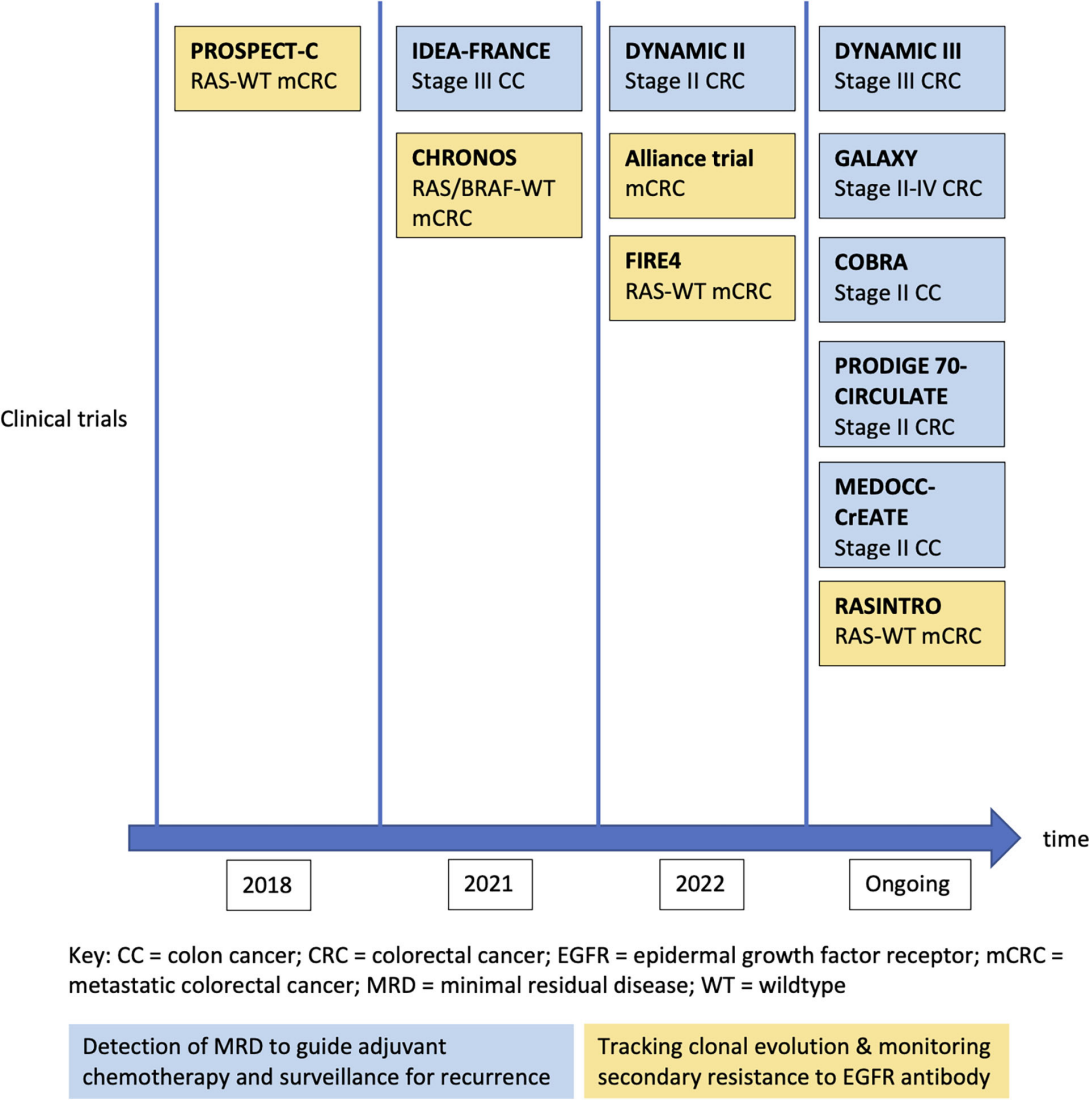
Development of major clinical trials for ctDNA application in colorectal cancers.

To date, the most mature clinical data on ctDNA are derived from patients with CRC. The detection rate of ctDNA in CRC is relatively high compared with other GI cancers that are discussed in this review - from an overall 73% in localized CRC (10), to 95.8% in patients with liver metastasis (16). **Figure 2** is a chronological overview of some of the key studies on the clinical application of ctDNA in the management of early and advanced CRC. Details of these studies will be discussed in the following sections.

### 3.1 Detection of minimal residual disease after surgery to guide adjuvant chemotherapy and surveillance for recurrence in early colorectal cancer

ctDNA allows the detection of MRD - residual cancer cells that are not detectable by conventional diagnostic tools (17). The current standard of care for stage III and some high-risk stage II colon cancers are surgery followed by adjuvant chemotherapy and then surveillance. The NCI Colon-Rectal-Anal Taskforce recommends the minimum time-points for perioperative sample collection to be 4-8 weeks post-resection, as cell damage during surgical resection and wound healing may lead to a surge in cfDNA (16).

#### 3.1.1 Earlier studies on prognostic significance of minimal residual disease after surgery

Earlier trials have consistently shown that ctDNA is a powerful prognostic biomarker for the early detection of recurrence in resectable CRC, independent of clinico-pathological factors. Several Australian studies have reported that the ctDNA detection rate in patients with stage II colon cancer following surgery is 7.9%; while the ctDNA detection rate in patients with stage III colon cancer following surgery is 21% (18, 19). In a cohort of 486 patients with stage II-III colon cancers and locally advanced rectal cancer (LARC), patients with detectable ctDNA (MRD+ve) after surgery experienced lower 5-year recurrence-free survival (RFS) rates of 38.6% versus (vs) 85.5% (P < 0.001) and overall survival (OS) rates of 64.6% vs 89.4% (P < 0.001), when compared with those patients with undetectable ctDNA (MRD-ve) (20). In addition, the risk of recurrence is proportionately increased with higher levels of ctDNA VAF (hazard ratio [HR] = 1.2, 2.5 and 5.8 for VAFs of 0.1%, 0.5% and 1%, respectively) (20).

A Danish trial showed that the risk of post-operative recurrence increases if ctDNA becomes first detectable at the following time-points: 7 times higher risk if soon after surgery, 17.5 times if detected after adjuvant chemotherapy, and 43.5 times during surveillance (21). Interestingly, the duration of adjuvant chemotherapy may affect the prognostic significance of ctDNA (22). In a *post-hoc* analysis from the IDEA-FRANCE trial (which investigated the optimal duration of adjuvant chemotherapy by comparing 6 months vs 3 months of treatment in stage III colon cancer), ctDNA was prognostic in patients who received 3 months of adjuvant chemotherapy and with T4 and/or N2 tumors, but not in those treated for 6 months and with T1-3/N1 tumors (22).

Besides investigating the prognostic significance of postoperative ctDNA status, other studies compared the performance of ctDNA and radiological imaging in the detection of post-operative recurrence. However, the results are mixed because of the different imaging intervals used across different studies (21, 23, 24). A Danish study showed that checking ctDNA every 6 months could detect cancer recurrences up to 16.5 months earlier than radiologic imaging (21). This result is supported by a prospective study using the Signatera assay (23), where ctDNA could detect recurrences at a median of 9.08 months earlier than imaging in 193 patients with stage II-III CRC. In the TRACC study (25), post-operative MRD+ve status was the most powerful prognostic factor associated with increased RFS (HR = 28.8; 95% CI = 3.5 - 234.1; P < 0.001), compared with clinical factors, microsatellite (MSI) and tumor mutational burden (TMB) in 122 patients with stage II-III CRC. In contrast, a retrospective study (n = 48, Signatera assay) found no significant differences in the lead time or rate of detecting postoperative recurrence between ctDNA and imaging - the latter performed at intervals recommended by the United States (US) National Comprehensive Cancer Network (NCCN) guideline (24). The ongoing observational study (BESPOKE) will evaluate the impact of ctDNA testing (Signatera assay) on adjuvant treatment decisions and detection of recurrence in stage I-IV CRC across over 200 US sites (26). In conclusion, these observational studies have shown that ctDNA could accurately detect MRD status after surgery and predict disease recurrence, thus prospective randomized trials are warranted to determine if ctDNA will influence treatment decisions.

#### 3.1.2 Recently reported phase III studies

There are at least 7 ongoing phase III studies with an interventional, observational or adaptive-platform design. These studies investigate the utility of postoperative MRD detection using ctDNA in guiding de-escalating or escalating adjuvant approaches in the management of stage II-III and/or resectable stage IV CRC (**Table 1**).

**Table 1:** Ongoing phase III or large observational studies or abstract-only reports on minimal residual disease.

Study name & design. First author & reference	Study population	Sample size	Assay	Timepoints of ctDNA analysis	Primaryendpoint	Preliminary results reported in abstract form
TRACC (NCT04050345) Phase II/III. Anandappa G, et al. (25)	High risk stage II, stage III CRC	107	Tumor- informed Multiplex PCR (Signatera)	Before surgery or nCRT, <8 weeks and 3 months post-surgery.	3-year DFS	-Baseline: 100/107 pts (93.4%) = ctDNA+ve -After treatment: 14/107 pts (13%) = MRD +ve; -6/14 pts (42.9%) MRD +ve relapsed vs 8/93 pts (8.6%) MRD-ve, -ctDNA status most significant prognostic factor associated with RFS
GALAXY study (UMIN000039205) Prospective observational study. Shirasu H, et al. (27)	Stage II-III, resectable stage IV CRC	1040	Tumor- informed Multiplex PCR (Signatera)	Before surgery, 4,12,24,36,48,72,96 weeks post-surgery	DFS	-188/1040 pts (18%) MRD+ve at 4 weeks 1-year DFS 47.5% in MRD+ve pts, vs, 1-year DFS 92.7% in MRD-ve pts.
VEGA study (jRCT1031200006) Phase III, non-inferiority study	ctDNA-ve pts at 4 weeks after surgery, high-risk stage II, low risk stage III CRC	1240	Natera, Inc, (bespoke, mPCR-NGS)	Postoperative week 4, then 3months after completing adjuvant chemo	DFS in ctDNA-ve pts randomized to surgery alone vs adjuvant CAPOX	Not available
ALTAIR study (NCT04457297) Phase III	Stage II-III or resectable Stage IV CRC who remain ctDNA+ve within 3 months after surgery and had adjuvant chemo	240	Tumor -informed Multiplex PCR (Signatera)	Postoperative and monthly up to 3 months	DFS in ctDNA+ve pts despite prior adjuvant chemo, randomized to trifluidine/tiparicil or placebo	Not available
DYNAMIC-III (ACTRN-12617001566325) Phase II/III	Stage III CRC	1000	Safe-SeqS	Week 5 to 6 postoperatively, then at end of adjuvant chemo	RFS for ctDNA +ve cohort and ctDNA-ve cohort, disease managed with escalated (if ctDNA +ve) de-escalated treatment (if ctDNA -ve)	Not available
DYNAMIC-RECTAL (ACTRN-12617001560381)	Locally advanced rectal cancer	408	Safe-SeqS	Week 4 and 7 post-op	RFS for ctDNA and pathology- guided treatment and standard of care	Not available
MEDOCC-CrEATE (NL6281/NTR6455) Phase III	Stage II colon cancer	1320	PGDx elio™	Immediate post-operatively in intervention arm, end of trial in control arm	Proportion of pts receiving adjuvant chemo when ctDNA+ve after surgery	Not available
PRODIGE 70 CIRCULATE (NCT04120701) Phase III	Resected stage II colon cancer	1980	ddPCR (2 methylated markers WIF1 and NPY)	≥ 2 weeks and <8 week postoperatively	ctDNA +ve cohort: 3-year DFS	Not available
COBRA (NCT0406810) Phase II/III	Resected stage IIA colon cancer	1408	Guardant Health LUNAR model	Post-operatively	Phase II subset: clearance of ctDNA for ctDNA+ve pts at baseline with/without adjuvant chemo ≤ 6 months from baseline. Phase III subset: RFS in ctDNA+ve cohort randomized to with/without adjuvant chemo	Not available
PEGASUS (NCT04259944) Phase II	Resected T4N0 or stage III colon cancer	140	Guardant LUNAR-1™	2-4 weeks after surgery, then 3 monthly or after each treatment	Number of post-surgery and post-adjuvant chemo false-negative cases after a double ctDNA-negative detection	Not available
CIRCULATE AIO-KRK-0217 (NCT04089631) Phase II	Stage II colon cancer	4812	Not reported	≤ 5 weeks postoperatively	ctDNA+ve: DFS in pts randomized to surgery alone or adjuvant chemo	Not available
BESPOKE (NCT04264702) Case-control study	Stage I-IV colon cancer	2000	Tumor -informed Multiplex PCR (Signatera)	Serially sampling post operatively up to 2 years	Impact of ctDNA on adjuvant treatment decisions Determine rate of recurrence of pts diagnosed with CRC while asymptomatic using ctDNA	Not available

CRC, colorectal cancer; PCR, polymerase chain reaction; DFS, disease free survival; MRD, minimal residual disease; +, positive; -, negative; RFS, relapse or recurrence-free survival, ddPCR, digital droplet polymerase chain reaction; Pt, patients; nCRT, neoadjuvant chemoradiotherapy; chemo, chemotherapy.

Several trials are investigating whether ctDNA-detected MRD can outperform conventional methods of directing adjuvant chemotherapy (**Table 1**). The DYNAMIC II (28) and III (ACTRN-12617001566325) studies are interventional studies led by the Australasian Gastro-Intestinal Trials Group for patients with stage II to III CRC. The pivotal DYNAMIC II trial is the first of these studies to be published recently, where 455 patients with stage II colon cancer (T3 or T4, N0, M0) were randomized in a 2:1 ratio to have treatment decisions guided by either ctDNA results (using Safe-Seq assay in a central laboratory) or standard clinicopathological features (29). Patients with a positive ctDNA result at either 4-week or 7-week after surgery received adjuvant fluoropyrimidine or oxaliplatin-based chemotherapy. Designed to detect non-inferiority between the 2 arms, the study met its primary endpoint by showing that ctDNA-guided approach was non-inferior to standard management (93.5% and 92.4% respectively; 95% CI = 4.1 - 6.2 [non-inferiority margin, −8.5% points]) in terms of 2-year RFS. Moreover, fewer patients in the ctDNA-guided arm received adjuvant chemotherapy (15% vs 28%; relative risk = 1.82; 95% CI = 1.25 - 2.65). This study is the first to show that ctDNA-guided approach to the management of stage II colon cancer could reduce the use of adjuvant chemotherapy use without compromising RFS. There are several other ongoing trials which investigate the utility of ctDNA in guiding adjuvant decisions. These include the PEGASUS study (NCT04259944, uses the LUNAR1 assay, Guardant Health) which has a novel, real-time adaptive design, where patients with stage II-III CRC will switch chemotherapy regimens based on the MRD status monitored at 3-month intervals (30). The US NRG-led COBRA (NCT04068103) is an escalation trial for stage II colon cancer, where MRD+ve patients will receive adjuvant chemotherapy, while MRD-ve patients will undergo surveillance alone (31). The French PRODIGE 70-CIRCULATE (NCT04120701) study will screen over 2600 patients with stage II CRC and randomize 198 MRD+ve patients post-surgery to either adjuvant FOLFOX (infusional 5-fluorouracil, leucovorin and oxaliplatin) for 6 months or observation. Similar to the DYNAMIC studies, the MEDOCC-CrEATE study will enroll 1320 stage II colon cancer patients without indication for adjuvant chemotherapy based on current practice guidelines, and randomize them into two possible interventional arms: ctDNA-uninformed (standard observation without adjuvant chemotherapy) vs ctDNA-informed (adjuvant CAPOX or observation, depending on MRD status) (29).

The largest study to date is the colossal ‘CIRCULATE-Japan’, an adaptive platform study which investigates the utility of ctDNA in MRD detection for patients with resectable stage II to IV CRC *via.* Eligible patients are first enrolled into an observational screening study (called GALAXY) and undergo ctDNA testing (Signatera assay) before treatment and at defined intervals after surgery. Each participant’s ctDNA results are made available to the treating physicians to guide adjuvant treatment or enrollment into either one of two interventional phase III studies – the VEGA and ALTAIR (32). Preliminary result of the GALAXY study on the ctDNA dynamics of 1040 (out of a target of 5200) patients have been reported (27, 33). For all stages of CRC, patients who were MRD+ve at 4 weeks post-surgery (18%, 188/1040 patients) had 1-year disease-free survival (DFS) rate of 47.5%; while patients who were MRD-ve at 4 weeks post-surgery (82%, 852/1040 patients) had a 1-year DFS rate of 92.7% with a HR of 10.9 (95% CI = 7.8 - 15.4; P < 0.001). Adjuvant chemotherapy may be able to convert patients who were initially MRD+ve into MRD-ve. The use of adjuvant chemotherapy resulted in a higher proportion of patients (68%) being converted to MRD-ve at 12-weeks post-surgery, while only 10% of patients who did not receive chemotherapy were converted into MRD-ve. Patients who were successfully converted from MRD+ve to MRD-ve status had better DFS. In contrast, in patient subgroups who were MRD-ve at 4 weeks post-surgery, the use of adjuvant chemotherapy did not influence the DFS (HR = 1.3; 95% CI = 0.5 - 3.6%). In conclusion, this study supports the use of ctDNA at 4 weeks post-surgery to guide adjuvant therapy. The ongoing ALTAIR study will randomize patients who are ctDNA+ve at any time-point within 2 years after curative-intent surgery, to 6 months of oral trifluridine/tipiracil or placebo. The VEGA trial investigates a de-escalation strategy of randomizing patients who were MRD-ve at 4 weeks post-surgery to either surgery alone or 3 months of adjuvant CAPOX.

### 3.2 Use of ctDNA in guiding treatment of locally advanced rectal cancer during preoperative chemo-radiotherapy

LARC is usually treated with multimodality treatment with either concurrent chemoradiotherapy (chemo-RT) or total neoadjuvant therapy (TNT) followed by total mesorectal excision surgery (TME). In general, ctDNA can be detected in approximately 57 - 77% of patients before surgery, 15.6 - 22.3% of patients after neoadjuvant therapy and 10.5 - 12% of patients after surgery - allowing for differences in the patients and assays across studies (34–36). Sampling of ctDNA at any of these perioperative time-points are also prognostic to a different extent. Appelt et al. (37) found that patients with baseline detectable hypermethylated ctDNA predicted improved OS (HR = 2.08; 95% CI = 1.23 - 1.51) and freedom from distant metastases (HR = 2.20; 95% CI = 1.19 - 4.07). Zhou et al. (34) showed that median VAF in baseline ctDNA was a strong independent predictor of metastasis-free survival (MFS) (HR = 1.27; P < 0.001). However, another study showed no association between ctDNA at baseline and MFS (38).

There is increasing interest in organ preservation strategies to spare patients the morbidity of a TME surgery. The recently reported phase II OPRA trial (NCT02008656) showed that up to half of patients may be able to achieve a clinical complete response (cCR) with a TNT approach without a detriment to DFS. ctDNA may complement conventional approach of predicting response to neoadjuvant therapy. Wang et al. (39) constructed a risk model unifying baseline ctDNA, ctDNA clearance, tumoral mutation status and magnetic resonance imaging (MRI)-based tumor regression grade in the prediction of pathological complete remission (pCR) after neoadjuvant therapy. This model was shown to be more accurate than models that were derived from only ctDNA or only MRI-based tumor regression grade.

The role of adjuvant chemotherapy after chemo-RT and surgery is controversial, with only one study showing a progression-free survival (PFS) benefit with FOLFOX in stage III LARC (40). Patients with detectable ctDNA after surgery are significantly associated with worse RFS if ctDNA is detected as early as 4-6 weeks after neoadjuvant chemo-RT (HR = 6.6; P < 0.001) or as late as 4-10 weeks after surgery (HR = 13.0; P < 0.001). This prognostic significance of MRD+ve status is independent of adjuvant chemotherapy and clinicopathological risk factors (36). This finding has formed the basis of the ongoing DYNAMIC RECTAL study (ACTRN-12617001560381) which will randomize patients to chemotherapy or surveillance after surgery, depending on the MRD status.

In conclusion, ctDNA has the potential of directing adjuvant therapy and improving the accuracy of assessing response to neoadjuvant therapy. These in turn may help to identify patients who might be candidates for de-escalated approaches, such as surveillance alone after TME, sphincter-preserving surgery or a wait-and-watch approach without surgery after chemo-RT. Validation in larger prospective trials using a risk-adapted approach in the management of LARC are ongoing.

### 3.3 Monitoring response to palliative chemotherapy in metastatic colorectal cancer

In stage 4 CRC, ctDNA VAF is significantly associated with the number of metastatic sites (41) and is prognostic in resectable or unresectable metastatic CRC (42). Chemotherapy and targeted therapy are part of the standard treatments for stage 4 CRC, and various biomarker-guided therapies targeting *BRAF* mutation and *EGFR*-mediated signaling have improved patient survival (3). Many studies have reported a high concordance between tumor and plasma in the detection of *KRAS* and *BRAF* mutations using ddPCR (43, 44). For patients with resectable oligometastatic CRC, detectable levels of ctDNA after surgery and/or post-operative chemotherapy are associated with shorter RFS (45).

The dynamic changes of ctDNA during the first few cycles of chemotherapy may predict radiologic response. In a study by Tie et al., 74% of patients had a 10-fold decrease in ctDNA level before cycle 2, which correlated with radiologic responses at 8-10 weeks (odds ratio [OR] = 5.25; 95% CI = 1.38 – 19.93; P = 0.016) (46). Conversely, an increase in ctDNA during the first cycle of chemotherapy could predict inferior outcome (47). The findings by Tie et al. are supported by another prospective study by Garlan et al., where ‘ctDNA responders’ had superior radiologic response, PFS and OS than those who were ctDNA non-responders (48).

### 3.4 Tracking clonal evolution and monitoring secondary resistance to epidermal growth factor receptor (*EGFR*) antibody

#### 3.4.1 Clonal dynamics during *EGFR* therapy alone or in combination with chemotherapy

In clinical practice, *RAS* and *BRAF* mutations are routinely analyzed to guide anti-*EGFR* therapy in mCRC. Other molecular alterations also contribute to resistance to *EGFR* antibody therapy, such as *PIK3CA* mutation, *HER2*, *MET* and *ERBB2* amplifications (49–51). Several landmark studies have suggested that when cancer cells are subjected to therapeutic pressure during anti-*EGFR* therapy, they acquire secondary genetic alterations in a process known as clonal evolution, which may contribute to drug resistance. Emergence of resistant clones can be tracked serially using ctDNA during anti-*EGFR* therapy, at as early as 10 months before the overt development of clinical resistance (52). Diaz et al. suggested that these drug-resistant *KRAS* mutant cancer cells are already present before a patients is started on *EGFR* antibody treatment (53). To confirm these findings, the PROSPECT-C phase II study was carried out to track the clonal evolution of resistant subclones using ctDNA during *EGFR* antibody therapy in patients with *RAS*-wildtype (WT) CRC. At baseline, 50% of patients already harbored aberrations in *RAS* pathway and *BRAF V600E* mutations in their ctDNA (54), and most patients (86.3%) would have detectable ctDNA levels of these resistant mutations at clinical progression (54).

ctDNA has also been used to track clonal evolution in treatment-naïve patients receiving *EGFR* antibodies together with chemotherapy. The CALGB/SWOG 80405 (Alliance) (55) is a first line study which randomizes patients to two different drug sequences of first-line therapy for stage IV CRC: chemotherapy plus *EGFR* antibody (cetuximab) or chemotherapy plus *VEGF* antibody (bevacizumab). In the *post-hoc* analysis of 133 patients with *RAS/BRAF*-WT CRC, ctDNA tracking showed a trend towards a higher prevalence of acquired mutations associated with resistance to *EGFR* antibody in patients (n = 11; 15.3%) randomized to the bevacizumab arm than patients in the cetuximab arm (n = 5; 8.2%) (OR = 2.0; P = 0.29). These provocative findings seem to suggest that exposure to bevacizumab in the first-line setting may increase the chance of patients acquiring *EGFR* antibody resistance-associated genomic alteration, thus further validation is warranted.

#### 3.4.2 *EGFR* antibody rechallenge

ctDNA is useful in selecting patients for *EGFR* antibody rechallenge. Clonal evolution is a dynamic process and therefore the optimal time-point and ctDNA VAF thresholds for determining whether a patient could be re-challenged with *EGFR* antibody therapy need to be defined. Siravegna et al. reported that the circulating level of mutant *RAS* clones increase initially during anti-*EGFR* therapy and gradually fall when therapy was withdrawn (49). Furthermore, circulating *RAS* and *EGFR* VAF undergo exponential decay at cessation of *EGFR* antibody therapy with a cumulative half-life of around 4.4 months (56). In a cohort of 80 patients who were re-treated with *EGFR* antibody, an overall response rate (ORR) of 23% was observed. A non-statistical trend towards higher ORR and PFS was noted if these patients were re-challenged after a longer drug holiday (in terms of <1 vs 2 half-lives) from the last *EGFR* antibody therapy (56). This knowledge may provide insight into the optimal timing of *EGFR* antibody re-challenge, however, the most appropriate VAF threshold that can guide treatment remains unclear (16). A meta-analysis showed that in patients without detectable *RAS* mutation in ctDNA, re-challenge with *EGFR* antibody therapy was associated with a larger benefit in PFS (HR = 0.40; 95% CI = 0.22 - 0.70; P = 0.001) and OS (HR = 0.37; 95% CI = 0.16 - 0.85; P = 0.02) than in patients with detectable *RAS* mutation (57). Several trials investigating the clinical impact of ctDNA-guided re-challenge of *EGFR* antibody therapy are ongoing, these include the RASINTRO (NCT03259009), FIRE4 trial (58) and the CHRONOS study (59) (**Table 2**). The CHRONOS is an interventional study which enrolls responders to *EGFR* antibody therapy who are ‘triple wild-type’ in *RAS*, *BRAF* and *EGFR* ectodomain in ctDNA. Patients will be re-challenged with panitumumab while ddPCR and NGS are used to track clonal evolution. Of the 52 patients screened in a preliminary report, 36 (69%) were triple wild-type, 27 received panitumumab with an ORR of 30% (59).

**Table 2:** Studies evaluating circulating tumor DNA as a screening tool to detect patients who could benefit from EGFR antibody re-challenge in metastatic colorectal cancer.

Study name	Study design	Estimated sample size	Assessment method/Assay	Mutation analyzed	Primary outcome	Secondary outcome	Results (abstract only)
CHRONOS (NCT03227926)Sartore-Bianchi A, et al. (59)	Phase II RCT	52	ddPCR, NGS	*RAS/EGFR/BRAF*	ORR	PFS, OS, Toxicity	-36/52 pts (69%) negative for *RAS/BRAF/EGFR* mutations. -ORR for rechallenge EGFR antibody = 30%
RASINTRO (NCT03259009)	Prospective observational cohort	73	NGS	*RAS*	PFS	Tumor response, OS	Not available
FIRE-4 (NCT02934529)	Phase III RCT	550	Not available	*RAS*	OS	PFS, ORR, molecular biomarker	Not available
PULSE (NCT03992456)	Phase II RCT	120	NGS (Guardant 360)	*RAS*	OS	PFS, ORR, CBR	Not available
COLOMATE (NCT03765736)	Prospective observational cohort	500	NGS (Guardant 360)	RAS/ERBB2/BRAF	Proportion of patients with an actionable genomic profile	Not available	Not available

RCT, randomised controlled trial; ddPCR, digital droplet polymerase chain reaction; NGS, next generation sequencing; ORR, overall response rate; PFS, progression free survival, OS, overall survival; CI, confidence interval; CBR, clinical benefit rate.

#### 3.4.3 Other targeted therapies

For other rarer molecular subgroups such as *BRAF* mutant, *HER2* amplified and MSI-high (MSI-H) CRC, newer drug therapies are becoming available in the clinic (3). ctDNA monitoring has been used to track clonal evolutions in patient subgroups with *BRAF V600E* mutations and *HER2* alterations in some clinical trials. In an exploratory analysis of the phase III BEACON trial (60) which demonstrated the superiority of targeting *BRAF-EGFR-MEK* inhibition with encorafenib-binimetinib-cetuximab over cetuximab-chemotherapy, over 90% of patients had detectable *BRAF V600E* mutations in ctDNA (GuardantOMNI assay). ctDNA VAF was found to be prognostic but not predictive of drug response (61). Patients with low ctDNA VAF (defined as lower than the median VAF) had longer median OS (14.8 months; 95% CI = 11.7 – 23.0) than in those with higher VAF (5.4 months; 95% CI = 4.4 – 6.1) when treated with encorafenib-cetuximab (61). In the phase II TRIUMPH trial (UMIN000027887) which tested the combination of trastuzumab plus pertuzumab in patients with *HER2*-amplified stage IV CRC, both tissue and ctDNA were used for determining *HER2* status. The ORR were similar in patients who were tested *HER2*+ve with tissue compared with ctDNA (ORR = 30% vs 28% respectively) (62).The COLOMATE study (NCT03765736) is an ongoing, seamless adaptive protocol that primarily uses ctDNA (Guardant 360) to screen patients with secondary resistance to targeted therapies, for enrolment into 3 different clinical trials depending on their ctDNA genotype: panitumumab re-challenge (PULSE study NCT03992456); tucatinib, trastuzumab, and TAS-102 for patients if ctDNA show *HER2*-alteration; and re-challenge with encorafenib, cetuximab and binimetinib if patient is *BRAF V600E*-mutant (63).

## 4 Gastric and esophageal cancer

### 4.1 Gastric and gastroesophageal junction adenocarcinoma

The application of ctDNA in gastric cancers (GC) remains challenging due to the relatively low frequency of genomic alterations, the larger inter-patient and intra-patient temporo-spatial heterogeneity in tumors and plasma, as well as impaired tumor shredding from peritoneal metastases (64–66). Around 37% of GC tumors contain actionable somatic mutations (e.g. *KRAS*, *TP53*, *PIK3CA*) or gene amplifications (e.g. *HER2*, *MET*, *EGFR*, *FGFR2*, *ERBB2*) (64, 67–69). Ichikawa et al. found that 68.1% of cancer-related genes identified in ctDNA of patients with GC are actionable, with *TP53* mutation and *ERBB2* being the most common (70). Maron et al. reported that in a large cohort over 1600 patients with GEA, the presence of some actionable *RTK* amplifications (e.g. *HER2*, *EGFR*, *MET*, *FGFR2*) are of prognostic significance (64).

Several comparative analyses of genomic profiling using ctDNA and tumors have been carried out in GC. The Korean VIKTORY trial of stage IV GC reported a 89.5% concordance between liquid and tumor biopsy for *MET* amplification (71). Schrock et al. reported a 86% concordance in genomic alterations detected in tissue and plasma derived from 417 patients with GI cancers; however, only 63% of alterations found in ctDNA were detected in tumor, suggesting intra-tumoral heterogeneity (69). Moreover, the concordance rate was lower (50%) for gene amplifications such as *HER2*. Studies on the concordance between *HER2* amplification in tumors using conventional methods (immunohistochemistry [IHC], or FISH) and ctDNA have shown mixed results. Some studies found high concordance with ddPCR (72, 73), but another showed that only 62% of patients with known *HER2*+ve tumors had detectable *HER2* amplification in ctDNA (64). In conclusion, these studies suggest that genotype information from ctDNA is complementary but cannot replace tumor-based NGS in GC (64).

#### 4.1.1 Minimal residual disease detection post-surgery

Similar to CRC, ctDNA has been investigated in the detection of MRD detection in resectable GC, gastroesophageal junction (GEJ) and esophageal adenocarcinoma (EAC). Data are limited by the relatively low level of ctDNA found before surgery (42 - 47%) in GC or EAC (74, 75). In one of the largest study in GEA, Maron et al. evaluated the utility of a commercial ctDNA-NGS assay (Guardant 360) 1630 patients with GC and EACs. MRD detection after curative surgery of EACs is strongly associated with an increased risk of recurrence (64). Kim et al. found that postoperative MRD+ve status in stage I-III GC precedes radiographic progression by 6 months (76), and is associated with shorter DFS (HR = 14.78; 95% CI = 7.991 – 61.29; P < 0.0001) and OS (HR = 7.664; 95% CI = 2.916 – 21.06; P = 0.002) (75). Similar findings are also reported by Openshaw et al. in GEJ cancers with shorter RFS (HR = 3.7; P = 0.028) (77).

#### 4.1.2 CtDNA in patients with advanced gastric cancer undergoing systemic therapy

In patients with advanced GC undergoing systemic therapy, Maron et al. showed that the maximal tumor VAF (maxVAF) in ctDNA could reflect tumor burden, such that in patients with a baseline maxVAF level of > 0.5%, who experienced a ≥ 50% fall in the maxVAF level during the first 5 months of systemic treatment, had superior median OS of 13.7 vs 8.6 months than those who had not (HR = 0.3; 95% CI = 0.1 – 0.8; P = 0.02) (64). The role of ctDNA in tracking clonal evolution in patients undergoing trastuzumab or lapatinib-based therapy has been evaluated in another study, where ctDNA monitoring has revealed multiple alterations that are purportedly associated with secondary resistance to anti-*HER2* therapies, such as *MYC*, *EGFR*, *FGFR2* and *MET* amplifications (78), as well as *PIK3CA*, *ERBB2/4*, *NF1* and *KRAS Q61R* mutations (79, 80).

The PANGEA is the first reported prospective study using a biomarker-guided platform to individualize patients with stage IV GEA for systemic therapy (81). Pre-treatment tumor and ctDNA-based NGS target sequencing, IHC of programmed death receptor-1 ligand (PD-L1) expression, TMB and Epstein-Barr virus (EBV) status were used to stratify and assign patients to receive 1 out of 6 matched monoclonal antibody against *PD1*, *EGFR*, *HER2*, *FGFR2* or *VEGFR2* (81). The PANGEA met its primary endpoint with 45 of 68 (66%) patients alive at 12 months - exceeding the 50% historical control rate (81). The PLAGAST (NCT02674373*)* study is an ongoing non-interventional study which is aimed at evaluating the association of ctDNA dynamics with prognosis and response in patients with GC undergoing systemic therapy. The Oesophageal Cancer Clinical Molecular Stratification (OCCAMS) Consortium is leading an ongoing study of patients with resectable EAC where ctDNA will be performed (Signatera assay) during postoperative surveillance. A preliminary report on 12 patients showed that MRD+ve has a sensitivity and specificity of 100% in detecting early postoperative recurrence (82). Ococks et al. reported that ctDNA+ve patients have a longer median cancer-specific survival (10.0 months) than ctDNA negative patients (29.9 months) (HR = 5.55; 95% CI = 2.42 - 12.71; P = 0.0003) (83). Bonazzi et al. reported that detectable ctDNA variants in post-treatment plasma is associated with inferior disease-specific survival, and VAF increased with recurrence (84). In conclusion, these studies validate that ctDNA is prognostic for relapse and survival, and could be incorporated for risk stratification of patients for adjuvant chemotherapy escalation or de-escalation.

### 4.2 Esophageal squamous cell cancer (ESCC)

The mutational profile of ESCC is different from that of esophageal adenocarcinomas (EAC), but similar to that of other squamous cell cancers (85, 86). In a meta-analysis on sequencing methodologies including ctDNA analysis in ESCC, ctDNA assays have a relatively low sensitivity of 48.9% (29.4 - 68.8%), but high specificity of 95.5% (90.6 - 97.9%) for detecting recurrence post-surgery (87). The data on the utility of ctDNA in MRD detection in ESCC are mostly derived from small, retrospective studies. Two reports reported a decrease in ctDNA VAF in patients post-surgery (88, 89). In patients with localized ESCC undergoing neoadjuvant therapy, MRD+ve status post-treatment was associated with increased risk of tumor progression (HR = 18.7; P < 0.0001), distant metastases (HR = 32.1; P < 0.0001) and shorter disease-specific survival (HR = 23.1; P < 0. 0001) (14).

## 5 Pancreatico-biliary cancer

### 5.1 Pancreatic ductal adenocarcinoma (PC)

The genomic characterization of pancreatic cancer (PC) shows that somatic mutations of *KRAS*, *TP53*, *CDKN2A* are common (90). In a meta-analysis on 369 patients, *KRAS* mutation can be detected in ctDNA with a pooled sensitivity of 70% and specificity of 86% (91). However, one of the major limitations on the clinical applicability of ctDNA-NGS in PC is the low concordance rate of 31.9% in the tumor vs ctDNA-derived result (91). This may be due to the hepatic clearance of ctDNA released from the PC primary at the hepatic portal vein (92). Another limitation is the false-positive ddPCR results caused by benign conditions such as pancreatitis, therefore the additional use of methylation markers has been suggested to minimize this possibility (92, 93).

The prognostic value of ctDNA has been evaluated in a recent meta-analysis of 48 studies of over 3000 patients with different stages of PC. This study found that the detection of *KRAS* mutations *via* ctDNA has a negative impact on OS and PFS in PC (HR = 2.42; 95% CI = 1.95 - 2.99 and HR = 2.46; 95% CI = 2.01 - 3.00, respectively) (94). In localized PC, detection of ctDNA preoperatively is associated with poorer RFS (HR = 4.1; P = 0.002) and OS (HR = 4.0; P = 0.003) (95). This is consistent in another study, where ctDNA detection is associated with inferior RFS and PFS (HR = 2.27; 95% CI = 1.59 – 3.24; P < 0.001) and OS (HR = 2.04; 95% CI = 1.29 – 3.21; P = 0.002) (96). These studies suggest that MRD detection using ctDNA in the early postoperative period is prognostic in resectable PC (95, 97, 98), but may be affected by the use of neoadjuvant chemotherapy (99). Nevertheless, subsequent detection of ctDNA during surveillance strongly predicts recurrence with a 90% sensitivity and 88% specificity (99). The ongoing interventional phase III DYNAMIC-Pancreas study (ACTRN-12618000335291) in early-stage PC will evaluate the utility of ctDNA in guiding adjuvant therapy in resectable PC.

Most studies which investigated the potential of ctDNA in monitoring response to chemotherapy in advanced PC used *KRAS* genotyping, while a few targeted other clonal mutations. *KRAS* mutation can be detected in 36 out of 54 (67%) of patients with advanced PC (100). Collectively, several studies have shown that ctDNA increase tends to precede clinical progression as determined by imaging and serum Ca19.9 level by a few months (100–102). In a meta-analysis of studies on patients with detectable *KRAS* before treatment, conversion to undetectable *KRAS* after treatment is associated with better prognosis (94). ctDNA has also been used to track other cancer-specific mutations such as *TP53*, *APC*, *ATM*, *FBXW7*, *SMAD4*, *CDKN2A* and other variants (101, 103). *BRCA1/2* mutations can be found between 1-10% of PC and may predict response to PARP inhibitors in the palliative setting (104). A study has found a high degree of concordance in *BRCA* mutation detected in tissue and plasma (103). Larger studies are needed to test the feasibility of using ctDNA to select and monitor patients for *BRCA1/2* mutation and PARP inhibitor therapy. In conclusion, the development of ctDNA in monitoring response to systemic treatment is still at an early stage and requires further validation.

### 5.2 Biliary cancer - extrahepatic (EHCC), intrahepatic (IHCC) and gallbladder cancer

Most patients with cholangiocarcinomas (CC) - including IHCC, EHCC and gallbladder cancer, are usually diagnosed at an advanced stage where post-operative recurrence risk is high (105). *FGFR1-3* fusions and *IDH1/2* mutations can be found in 15 - 20% of IHCC, where the concordance of tumor and ctDNA-derived is higher for IHCC (92%) than that of EHCC (55%) (105). The detection of ctDNA using target-panel NGS has been used to track clonal evolution during chemotherapy, demonstrating that over 60% of patients may develop new driver genes at progression (105). There have been significant advances in the development of new targeted therapies for CC such as *IDH1* inhibitor for *IDH1* mutant tumors (106), and *FGFR* inhibitors for tumors harboring *FGFR2* fusions (107, 108). ctDNA has been investigated in selecting patients for such therapies and in tracking emergence of secondary resistance to these agents. Goyal et al. were the first to describe the molecular basis of acquired resistance to a *FGFR2* antibody (BGJ39) (109) by using serial cfDNA monitoring during treatment. An acquired *V564F* mutation was found in 3 out of 4 patients who progressed, while 2 progressors had multiple *FGFR* point mutations. There was a high concordance between tissue and ctDNA in detecting these resistant variants. This study may pave the way for larger studies on ctDNA in guiding anti-*FGFR2* therapy for CC.

## 6 Response monitoring of immune-checkpoint inhibitor therapy in GI cancers

Immune-checkpoint inhibitors (ICI) such as PD1 and CTLA-4 therapy are now part of the standard therapeutic options for stage IV MSI-H CRC in the first and subsequent line settings. In addition, patients with other GI cancers that are MSI-H or TMB > 10 mut/Mb (110) may be suitable for anti-PD1 therapy in the palliative setting. Several studies have investigated the feasibility of ctDNA in assessing MSI, TMB status in GI cancers. Nakamura et al. compared ctDNA NGS (Guardant 360) and tissue based MSI assessments in a cohort of 658 patients with advanced GI cancer in the SCRUM-Japan GOZILA study - an observational ctDNA-based study which screens patients with GI cancers for enrollment into clinical trials within a nation-wide trial network (111). The concordance between tumor and ctDNA for detection of MSI is high with an overall percent agreement of 98.2% (95% CI = 96.8 - 99.1). In particular, ctDNA was able to identify patients with MSI-high tumors who might benefit from anti-PD1 therapy (111). Using the Guardant360 assay, Maron et al. reported a 100% concordance between tumor-derived MMR status (IHC) and plasma-derived MSI-status using ctDNA-NGS in 6 patients (64). In contrast, there is significant discordance between tumor and ctDNA-derived TMB assessment. In a ‘real-life’ retrospective study of 410 patients (82 had GI cancers) with matching TMB results from tumor and plasma-based commercial NGS assays in the community setting, the median TMB was higher in plasma (*m* = 10.5 mut/Mb) than in tumor (*m* = 6.0 mut/Mb; P < 0.001). This will have obvious implication on selecting patients with non-CRC GI cancers for PD1 inhibitors based on ctDNA TMB alone, since the drug label for the tissue-agnostic approval of pembrolizumab recommends that the TMB threshold should be ≥ 10 mut/mb. In conclusion, if ctDNA TMB is used to select patients with GI cancers for PD1 inhibitor, a much higher ctDNA TMB threshold (up to 12 to 40 mut/Mb depending on the assays used) should be used to guide treatment decisions (112).

The monitoring of ctDNA during ICI therapy has been investigated in stage IV MSI-H CRC in a number of small cases reports. In these studies, the following endpoints were analyzed: the quantitation of VAF, measurement of TMB and tracking of tumor-specific mutations such as *TP53*, *RAS* and *BRAF* (113). Some studies have suggested that MSI-H CRC are often poorly-differentiated and produce significantly lower levels of serum tumor markers such as CEA and CA 19.9 (114–116) than well-differentiated tumors. Therefore, ctDNA holds promise as a blood-based predictive biomarker of response to ICI for such patients.

In Zhang et al.’s study of 978 patients across 16 tumor types (48 had GEAs, 32 had PC and 58 had MSI-H solid tumors) who were undergoing ICI therapy, changes in VAF during treatment could predict drug response, such that patients who could completely clear ctDNA (VAF = 0) had longer PFS and OS (P < 0.0001) than those who could not (117). Similarly, Kim et al. also found that in a study of 61 patients with stage IV GC treated with a PD1 inhibitor, changes in the ctDNA levels at 6 weeks post-treatment correlated with PFS and ORR (118).

Apart from VAF clearance as an endpoint, another study by Jin et al. investigated other endpoints e.g. ‘decline in maxVAF’ and ‘ctDNA-positivity’ *via* a NGS ctDNA assay, in 46 patients treated with PD1 inhibitor alone or in combination with chemotherapy (119). The median PFS was significantly longer in patients who experienced >  25% decline in maxVAF (7.3 months vs 3.6 months, P = 0.0011; 53.3% vs 13.3%, P = 0.06), and in those who had undetectable ctDNA (7.4 months vs. 4.9 months, P = 0.025) after ICI-based therapy (119).

## 7 Current challenges and future directions

Advances in NGS and PCR technologies have enabled the accurate detection and quantification of ctDNA in patients with different stages of GI cancers. There is a practical need to identify an informative and less invasive biomarker to help guide adjuvant, neoadjuvant and palliative drug therapies. The strongest evidence available to date showed that ctDNA is a strong prognostic marker when used to detect MRD following curative intent surgery in resectable GI cancer. The DYNAMIC II study is the first to show that ctDNA can direct adjuvant chemotherapy in the management of stage II colon cancer without compromising RFS. Several interventional studies with adaptive design using ctDNA as a screening platform are ongoing in patients with resectable CRC, PC and LARC. These studies are designed to definitively address the questions of whether ctDNA is superior to conventional methods of guiding adjuvant chemotherapy on patient’s survival, and whether ctDNA guided escalation or de-escalation of adjuvant therapy may help to improve survival and minimize the risk of long-term treatment-related morbidities.

In the palliative setting, there are emerging data to suggest that ctDNA dynamics during the early treatment period are both prognostic and/or predictive of subsequent response to systemic treatments. Furthermore, serial measurement of ctDNA during targeted therapies has enabled tracking of clonal evolution and emergence of secondary resistance-related variants to some targeted therapies. Some evidence supports the use of ctDNA in guiding *EGFR* antibody rechallenge followed by ICI, while more evidence is needed for other targeted therapies e.g. *HER2* or *BRAF*.

As NGS technologies and other pre-analyzed variables are refined continuously with time, the cost and accuracy of ctDNA are likely to improve with time. There remain challenges that need to be overcome. It is unclear whether it is more informative to do both blood and tumor NGS at baseline than other modality alone in guiding treatment decisions or in selecting patients for clinical trials, given the intratumoral heterogeneity in GI cancers. Furthermore, there is a lack in consensus on determining the most biologically meaningful thresholds of ctDNA-related metrics (e.g. maxVAF, percentage change in VAF) to guide oncologists in practice. In postoperative surveillance, it is unlikely that ctDNA will completely replace conventional diagnostic and staging tools e.g. imaging and protein-based serum cancer markers in the management of GI cancers. In clinical trials, ctDNA may potentially accelerate drug development by facilitating the molecular genotyping of patients for clinical trials of novel targeted therapies, in detecting early signals of drug response and in tracking emerging clonal resistance (120).

In conclusion, it is important to reach consensus on how ctDNA as a biomarker should be practically incorporated into current complex treatment algorithms to guide the treatment of GI cancers in potentially curative and palliative settings. One of the possible directions is to use the massive volume of genomic data derived from the systemic profiling of ctDNA for the development of artificial intelligence driven computational models and programs that can be applied in the routine oncological care of patients with GI cancers.

## Author contributions

RL and BM are responsible for overall design, planning, writing and editing of manuscript and figure. DJ is responsible for writing up of specific sections and the table. WL, KC, GL, ML, and JW are responsible for planning and editing of specific sections. All authors contributed to the article and approved the submitted version.

## Funding

This review is supported in part by the Kingboard Precision Oncology Program and the Charlie Lee Precision Immuno-oncology program, The Chinese University of Hong Kong, Hong Kong SAR.

## Acknowledgments

We would like to thank Ms. Alice Kong for clerical assistance.

## Conflict of interest

KC is a director of Take2, DRA and Novostics. KC holds equities in Take2, DRA, Grail/Illumina. KC and WL were previous consultants to Grail. WL holds equity in Grail/Illumina. KC holds patents portfolio in molecular diagnostics and receive royalties from Take2, DRA, Grail, Illumina, Sequenome, Xcelom.

The remaining authors declare that the research was conducted in the absence of any commercial or financial relationships that could be construed as a potential conflict of interest.

## Publisher’s note

All claims expressed in this article are solely those of the authors and do not necessarily represent those of their affiliated organizations, or those of the publisher, the editors and the reviewers. Any product that may be evaluated in this article, or claim that may be made by its manufacturer, is not guaranteed or endorsed by the publisher.
